# Efficacy and safety of repetitive transcranial magnetic stimulation in youth with depression: a systematic review and meta-analysis of randomized sham-controlled trials

**DOI:** 10.1007/s12519-025-00983-7

**Published:** 2025-10-25

**Authors:** Yu-Jie Tao, Xiao-Xia Duan, Pei Liu, Mei-Wen Wang, Si-Xun Li, Ting-Ting Luo, Hao-Yang Xing, Yi Huang

**Affiliations:** 1 Laboratory of Child and Adolescent Psychiatry, Mental Health Center and Psychiatric Laboratory, West China Hospital, Sichuan University, Chengdu 610041, China; 2 Department of Psychiatry, Child Mental Health Center, West China Hospital, Sichuan University, Wuhou No. 37, Guoxue Alley, Chengdu, China; 3 Department of Psychiatry, West China Second University Hospital, Sichuan University, Chengdu, China; 4 Huaxi MR Research Center (HMRRC), Functional and Molecular Imaging Key Laboratory of Sichuan Province, Department of Radiology, West China Hospital of Sichuan University, Chengdu, China; 5 School of Physical Science and Technology, Sichuan University, Chengdu, China

**Keywords:** Depression meta-analysis, Randomized sham-controlled trials, Repetitive transcranial magnetic stimulation, Youth

## Abstract

**Background:**

Major depressive disorder is a major cause of disability and health-related burden globally. Repetitive transcranial magnetic stimulation (rTMS) has emerged as a promising alternative therapy for major depressive disorder in adults, but its efficacy and safety in 10–25 years (youth) with depression remains inconclusive. We aim to evaluate the efficacy and safety of rTMS in youth with depression in randomized sham-controlled trials.

**Methods:**

A comprehensive search of nine databases was conducted from inception to April 30, 2025. Trials using random assignment with a sham control group were selected. Heterogeneity among studies was assessed using the *I*^*2*^ and Cochran *Q* test. A random-effects model was employed when *I*^*2*^ > 50%. Standard mean deviation (SMD) for depression rating scale scores and risk difference (RD) with corresponding 95% confidence intervals (CIs) of adverse event were used to evaluate efficacy and safety, respectively.

**Results:**

Sixteen studies with 1295 patients aged 10–25 years were included. Meta-analysis showed that active rTMS significantly reduced depression scale scores (SMD = – 0.93, 95% CI = – 1.31 to – 0.55). Subgroup analysis revealed significant relief of depressive symptoms at the second week (SMD =  – 0.66, 95% CI = – 1.25 to – 0.07) and persisting at the fourth week (SMD =  – 1.28, 95% CI = – 1.82 to – 0.75) when compared to sham stimulation. Pooled RR was 1.24 (95% CI = 1.06–1.45) for response rate and 1.63 (95% CI = 1.11–2.39) for remission rate (with an associated number needed to treat of 10).

**Conclusions:**

Evidence indicates that rTMS is effective, safe and exhibits a relatively rapid onset of action for treating youth depression. Larger-scale studies with longer treatment durations and extended follow-up periods are essential to understand and characterize the short- and long-term neuromodulatory effects within this vulnerable population. The effect of rTMS in treatment-resistant depression and its use across diverse populations also need further investigation.

**Graphical Abstract:**

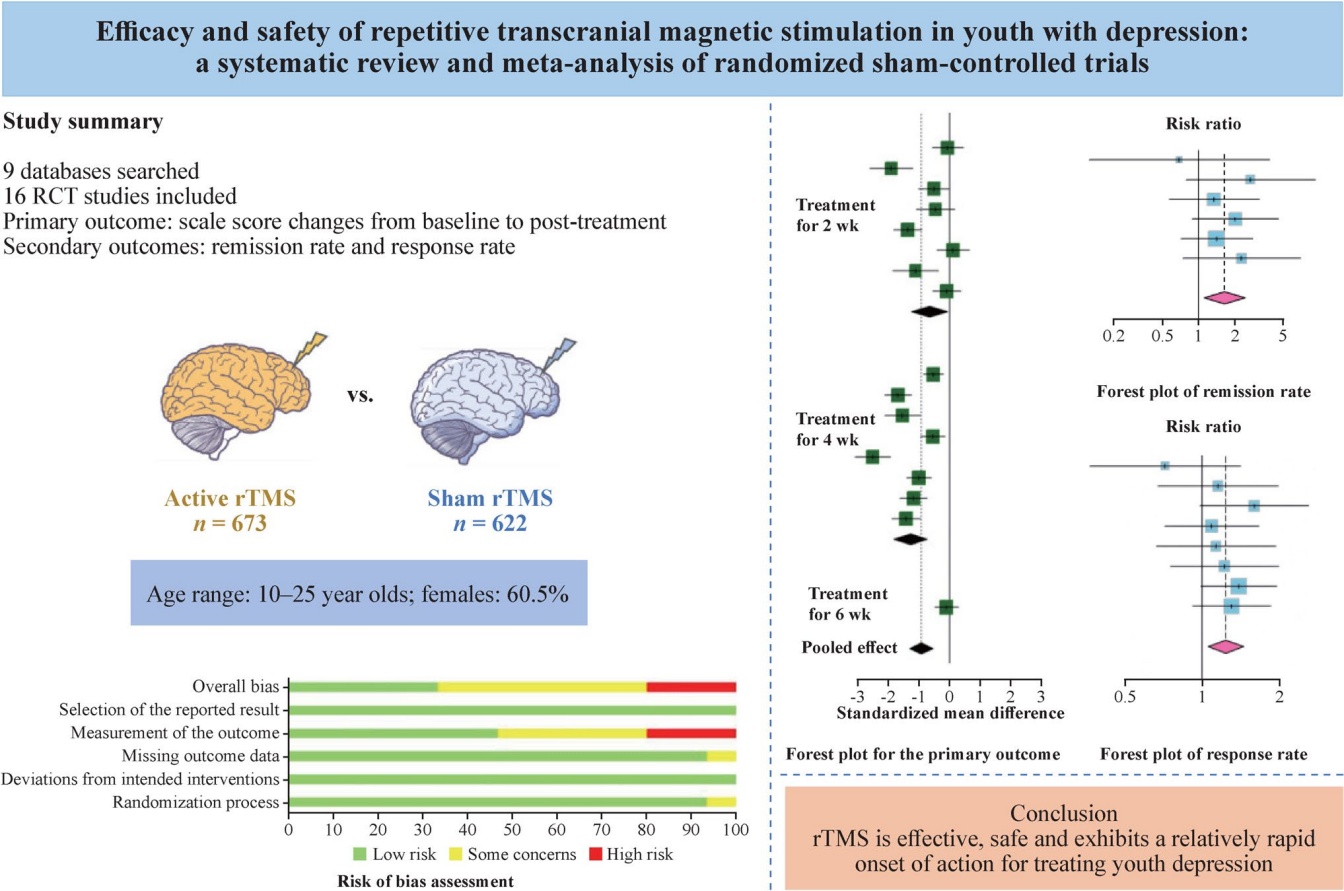

**Supplementary Information:**

The online version contains supplementary material available at 10.1007/s12519-025-00983-7.

## Introduction

Major depressive disorder (MDD) is recognized as the principal cause of disability and health-related burden globally, particularly among 10–25 years (youth) [1, 2]. According to the 2019 Global Burden of Disease (GBD 2019), years of disability lost (YLDs) due to depressive disorders ranked second among all health conditions for adolescents aged 15‒19 years [3, 4]. The latest investigation reported that an estimated 1.37% of adolescents aged 10–14 years and 3.51% of those 15–19 years suffer from depression [5]. Adolescent-onset depression is associated with more frequent depressive episodes, longer duration episodes, increased comorbidities, suicidal ideation, poorer physical outcomes and impaired social functioning [6, 7]. Over the past two decades, pharmacological and psychological interventions have been widely employed to treat MDD in youth [8, 9]. Nonetheless, treatment outcomes remain unsatisfactory, with over 30% of patients failing to respond to initial treatment and up to 40% not responding to a second medication [10, 11].

Given these challenges, attention has turned to repetitive transcranial magnetic stimulation (rTMS) as a novel approach to treating depression in youth. rTMS is a non-invasive neuromodulation technique well-established in adult depressive disorder [12]. It utilizes electromagnetic fields targeted at the dorsolateral prefrontal cortex (DLPFC) to modulate neuronal and circuit activity involved in the regulation of emotion [13, 14]. Initially, Food and Drug Administration (FDA) approval limited this technique to adults with MDD [15], but accumulating evidence suggests efficacy and safety in adolescents and transitional youth with depression. Early findings from small-sample open-label trials indicate that high-frequency (10 Hz) rTMS may be a well-tolerated therapy for treatment-resistant adolescent depression, with mild to moderate and self‐limiting side effects [16–20]. Moreover, Wallman et al. found that both low-frequency (1 Hz) and high-frequency (10 Hz) rTMS are safe and well-tolerated, with a similar frequency and severity of adverse events [21]. Zhang et al. reported improved therapeutic efficacy of rTMS in adolescents compared to adults with MDD in a large naturalistic study [22]. In addition, a longitudinal study by Mayer suggested potential long-term benefit from the rTMS treatment sessions in youth [23].

In March 2024, the FDA approved rTMS for use in patients aged 15 and older [24]. However, the evidence primarily derives from open-label clinical trials that inadequately represent critical adolescent populations with high-risk profiles. Therefore, the application of rTMS in young people still warrants further exploration. Currently, systematic reviews and meta-analyses suggest that rTMS could reduce depressive symptoms and may offer a promising alternative therapy in young people [25–28]. Most existing meta-analyses are of open trials, lack strict sham-controlled randomized trials, have not included the latest research in the field and precluded the theta burst stimulation (TBS) mode that is currently widely studied. Moreover, no studies have explored the impact of medication usage on the therapeutic efficacy of rTMS. In addition, results from recent randomized sham-controlled studies are inconsistent. For instance, a previous large randomized double-blind sham-controlled trial involving 103 adolescents with treatment-resistant depression (TRD) found no significant change in depression severity scores post-stimulation between the active and sham intervention groups [29]. However, a study by Zhao et al. using a similar protocol of intermittent theta burst stimulation (iTBS) (triplet 50-Hz bursts repeated at 5 Hz, 2 seconds on, 8 seconds off, 1200 pulse/day,10 days), reported better overall efficacy in the active compared to the sham group [30].

Further research is clearly required to clarify the role of rTMS in treating depression in youth. There are several high-quality studies in this area that may have important implications for existing conclusions [30–36]. Our aim is to use meta-analysis to systematically review existing evidence from randomized sham-controlled trials to provide a more reliable interpretation of the efficacy and safety of rTMS in this population.

## Methods

The present study adhered to the Preferred Reporting Items for Systematic Reviews and Meta-Analyses (PRISMA) 2020 guidelines to ensure the review quality [37]. The study protocol was registered in the International Prospective Register of Systematic Reviews (PROSPERO) under the number CRD 42024549265. During PROSPERO registration we had initiated but not completed a pilot of the preliminary search and selection process. The reference to “12 of the 15 studies” in the data synthesis strategy was merely to illustrate our anticipated synthesis approach based on the initial pilot and did not represent final study selection or data analysis results. This was a prospective strategy designed to guide the synthesis process after all studies were selected. After registration and as the review process progressed, we ultimately confirmed 16 studies. Actual synthesis and analysis were carried out after registration and in strict accordance with the registered protocol.

### Search strategy and selection criteria

A comprehensive literature search was conducted across nine databases [PubMed, Embase, PsycINFO, Cochrane Library, Scopus, ProQuest Dissertations and Theses, Web of Science, China National Knowledge Infrastructure (CNKI) and WanFang] without restrictions on date, language, ethnicity or publication type. By the time of the analysis we had conducted two rounds of searches. The first round was from database inception until April 28, 2024, while the second round focused on literature published from April 28, 2024 to April 30, 2025. Our search utilized keywords including “rTMS, repetitive transcranial magnetic stimulation, TBS, intermittent theta burst stimulation, depression, depressive disorder, children, adolescent, youth, randomized controlled trials, RCT”. Full database search strings are detailed in the supplementary material. In addition, reference lists of included studies and previous relevant systematic reviews were manually screened for potentially relevant studies and efforts were made to obtain unpublished or missing data by contacting corresponding authors.

Adolescence is a transitional stage from childhood to adulthood and its definition has long been a matter of debate. According to the World Health Organization (WHO), the age range for adolescents is 10–19 years old (https://www.who.int/health-topics/adolescent-health#tab=tab_1). During our search, we found that many articles on rTMS treatment for adolescent depression included subjects over the age of 19. In addition, almost all meta-analyses on TMS treatment for adolescent depression included studies with participants aged 10–19 and older [25, 27, 38]. Moreover, recent work has expanded the definition and timeframe of adolescence to include young adulthood, often up to 25 years of age. This expansion is consistent with both a biological and sociological phenomenon known as the prolongation of adolescence [39, 40], and has also been proposed as an age range for “youth psychiatry” [41]. In summary, to better conform to the subjects of the included studies, we have limited the age range of our subjects to 10‒25 years old and defined this as youth.

Inclusion criteria were as follows: (1) participants aged 10‒25 years with diagnosis with MDD according to the diagnostic and statistical manual of mental disorders (DSM-IV/DSM-5) or depressive disorder according to the International Classification of Diseases, tenth edition (ICD-10); (2) intervention was rTMS or TBS, with or without antidepressants, with the control group receiving sham stimulation alone or combined with antidepressants, and (3) articles written in English or Chinese. Exclusion criteria were: (1) focus on other axis I disorders or alternative therapeutic approaches; (2) lack of primary outcome data on change in depression rating scale scores from baseline to post treatment; (3) use of other TMS modalities such as deep TMS (dTMS) or accelerated TMS (aTMS); (4) combination of rTMS or TBS with other non-pharmaceutical interventions, and (5) participants with serious or unstable medical conditions, including neurological, endocrine, and rheumatic conditions, brain disease, traumatic brain injury or surgery and infectious diseases.

Two independent researchers (TYJ and LP) screened identified records and selected final studies based on titles, abstracts and full-text. Discrepancies were resolved through discussion, with unresolved conflicts arbitrated by a third senior reviewer (HY) to reach consensus on study eligibility.

### Data extraction

Data from eligible studies were extracted using a predefined form. Two reviewers (TYJ and DXX) independently extracted relevant information, resolving disagreements through discussion or consultation with a third reviewer (WMW). Extracted study information included: first author’s name, publication year, sample sizes, gender distribution, age distribution and mean age of each treatment group, rTMS parameters (targets, stimulation frequency, number of sessions, percentage of resting motor threshold (%RMT), total number of pulses), treatment duration, time of onset to treatment and study design. The primary outcomes were defined in advance in the included studies and were determined by measuring the changes in the severity of depression from baseline to post-treatment using effective assessment scales such as the hamilton depression scale (HAMD), self-rating depression Scale (SDS) and baker depression inventory-II (BDI-II). Mean and standard deviation of scale scores at each assessment point (first week, second week, fourth week and sixth week) were extracted, along with response and remission rates, type and frequencies of adverse events reported in each study.

### Assessment of risk of bias in included studies

The Cochrane Risk of bias tool for randomized trials (ROB 2) [42] was used to assess the methodological quality of each trial; this was done independently by two investigators (TYJ and DXX). Any discrepancies were resolved through consensus within the review team. The risk of bias in each trial was categorized as “low”, “some concerns” or “high” risk of bias.

### Statistics

We conducted all data analyses using R software (version 4.4.0). Standardized mean differences (SMDs) were calculated for continuous outcomes, while risk differences (RDs) or risk ratios (RRs) were calculated for binary outcomes and their 95% confidence intervals (CIs) were reported. The primary outcome of treatment efficacy was severity of depression, assessed by SMDs between groups. The secondary outcomes of treatment efficacy were the response rate and remission rate. Safety was evaluated based on the incidence of adverse events during rTMS treatment. For those significant clinical event outcomes, we calculated the number needed to treat (NNT) for an additional beneficial outcome [43].

Heterogeneity among studies was assessed using the *I*^*2*^ statistic and Cochran Q test. If *I*^*2*^ > 50%, a random-effects model was used for meta-analysis. The possibility of publication bias was evaluated using a funnel plot and asymmetry was assessed using Egger’s test. Sensitivity analysis was performed by iteratively excluding individual articles.

Subgroup analyses were planned if substantial heterogeneity was detected or if there were at least two studies per subgroup. These analyses aimed to evaluate the potential impact of the following factors on the observed treatment efficacy: intervention intensity (treatment duration and rTMS frequency), depression severity (mild-moderate versus severe), pharmacological treatment before rTMS (treated versus untreated) and medication during intervention.

## Results

### Study characteristics

**Fig. 1:**
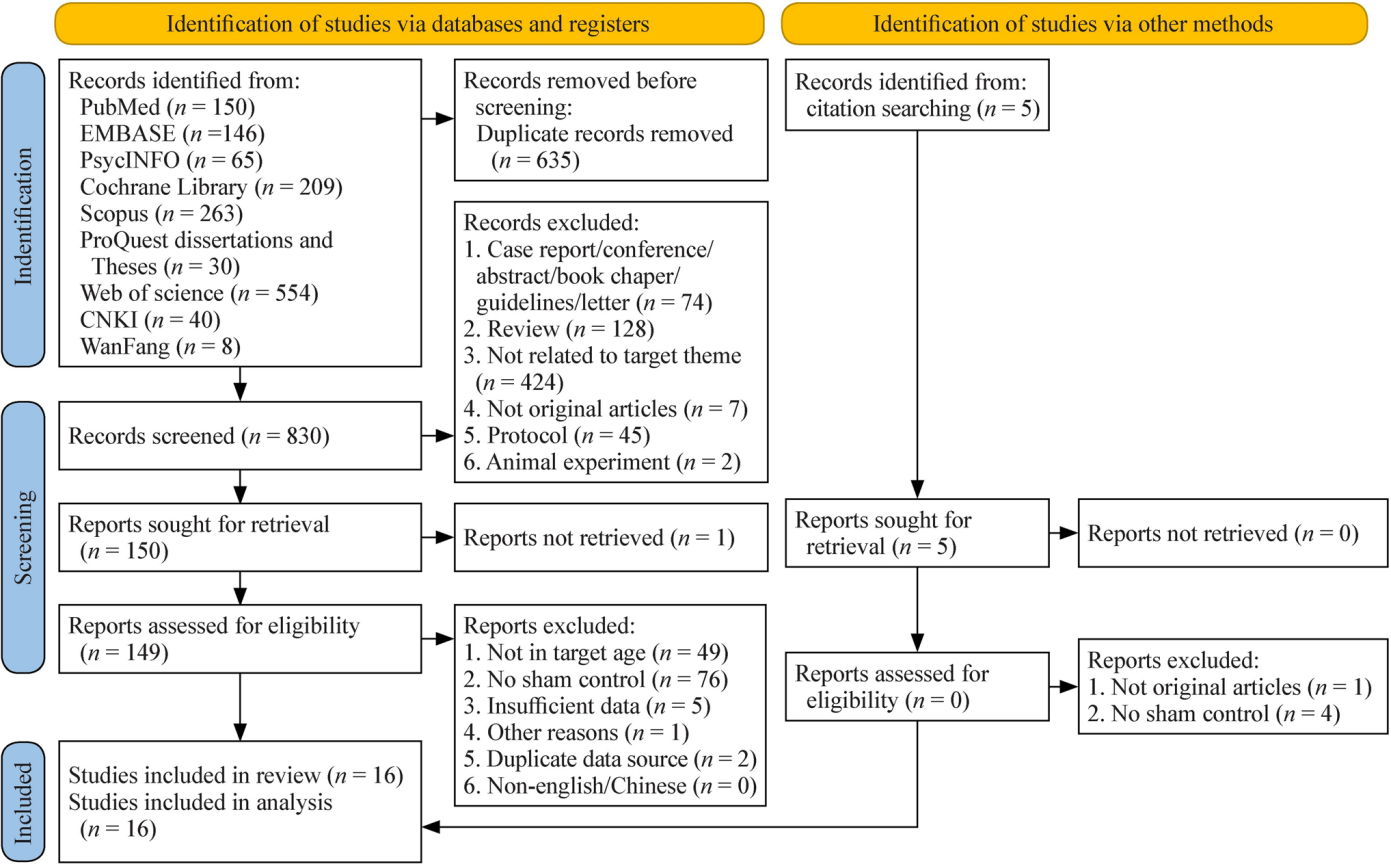
Preferred Reporting Items for Systematic Reviews and Meta-Analyses flowchart for study selection. *CNKI* China National Knowledge Infrastructure

Our database and hand searches initially identified 1465 studies, of which 830 remained after removing 635 duplicates. Following screening of titles and abstracts, 149 studies underwent full-text review and no eligible non-English/non-Chinese publications were identified that required progression to full-text retrieval. Amongst these studies, two utilized similar data sources, 76 articles lacked sham stimulation in the control group and 49 studies did not focus on youth. Five articles were excluded due to insufficient data on the primary outcome measures (one provided only remission and response rates [44]; one provided only the reduction in scale scores after treatment [45]; two provided only the mean scores of each dimension of the depression scales without a total score [46, 47], and one had a study population age range of 13–45 years without subgroup scale measurements for youth [48]). In addition, the study of Sun et al., was excluded because its design compared active rTMS targeting both the DLPFC and bilateral supplementary motor areas (SMAs) in the treatment group versus active rTMS stimulation of the DLPFC plus pseudo-bilateral SMA stimulation in the control group. This design fails to isolate the therapeutic efficacy of traditional DLPFC stimulation relative to sham conditions [49]. Ultimately, 16 studies met the inclusion criteria, encompassing 1295 participants (673 in the active stimulation group and 622 in the sham stimulation group) [30–36, 50–57]. Ten of these studies are newly included in this update compared to antecedent reviews [27, 28]. The selection process and reasons for exclusion are depicted in the PRISMA flowchart (Fig. 1), with detailed study characteristics provided in Table 1.

**Table 1:** Overall characteristics of included studies

Studies	Demographic					rTMS parameters							
	*n*	Age (y)	Male/female (*n*)	Cortical target	Frequency (Hz)	Sessions	%RMT	Total pulse	Pulse/session	Treatment duration (wk)	ROB domains (D1-D5)	Pharmacological treatment before rTMS	Medication during intervention
Zhang, et al., 2024^a^	61	I: 15.83 ± 2.41	46/13	Left DLPFC (BA46)	Triplet 50-Hz bursts repeated at 5 Hz	10	100	6000	600	2	+ + + + +	Treated	III
		C: 14.60 ± 2.85)											
Zhao, et al., 2023^a^	45	I: 17.13 ± 1.94	8/37	Left DLPFC (5 cm rule)	Triplet 50-Hz bursts repeated at 5 Hz	10	80	18,000	1800	2	+ + + ? +	Treated	I
		C: 17.29 ± 2.59											
Zou, 2023^b^	60	I: 17.53 ± 3.07	18/24	Right DLPFC (F4 site)	1 Hz	10	80	10,000	1000	2	+ + + ? +	Treated	I
		C: 17.17 ± 2.32											
Wang et al., 2023^b^	152	I: 14–16	70/82	Left DLPFC	10 Hz	20	90	40,000	2000	4	+ + + - +	Treated	II
		C: 14–16											
Lu and Gao, 2020^b^	108	I: 14.27 ± 2.31	51/57	Left DLPFC (F4 site)	10 Hz	20	80	12,000	600	4	? + + + +	Untreated	II
		C: 14.18 ± 2.29											
Qu et al., 2023^b^	60	I: 15.76 ± 1. 23	26/34	Right DLPFC	1 Hz	20	100	24,000	1200	4	+ + + + +	Treated	I
		C: 15.84 ± 1.28											
Gu, 2025^a^	40	I: 14.9 ± 2.1	13/27	Right DLPFC	1 Hz	10	90	15,000	1500	2	+ + + + +	Untreated	III
		C: 14.9 ± 1.7											
Chen et al., 2023^b^	100	I: 13.30 ± 1.43	20/80	Right DLPFC (F4 site)	1 Hz	20	50	10,000	1000	4	+ + + - +	Treated	II
		C: 13.36 ± 1.35											
Ran et al., 2022^b^	80	I: 14.89 ± 2.62	43/37	Left DLPFC	15 Hz	20	80	40,000	2000	4	+ + + - +	Untreated	II
		C: 14.56 ± 2.33)											
Liu, 2022^b^	90	I: 15.25 ± 3.99	37/53	Left DLPFC	10 Hz	12	120	36,000	3000	2	+ + + - +	Treated	II
		C: 16.50 ± 3.70											
Li, 2022^b^	55	I: 14–17, C: 14–17	21/34	Right DLPFC (5 cm rule)	1 Hz	10	80	10,500	1050	2	+ + + ? +	Treated	II
Fu et al., 2022^b^	104	I: 15.40 ± 2.41	29/75	Right DLPFC	1 Hz	20	100	40,000	2000	4	+ + + + +	Treated	III
		C: 15.49 ± 2.37											
Gao et al., 2021^b^	32	I: 17.00 ± 2.03,	9/23	Right DLPFC	1 Hz	10	80	10,000	1000	2	+ + + + +	Treated	II
		C: 17.00 ± 2.58											
Jiao et al., 2024^a^	135	LF: 15.07 ± 1.39	58/77	Right/left DLPFC	1/10 Hz	20	80/110	24,000	1200	4	+ + + ? +	Untreated	II
		HF: 14.87 ± 1.31											
		C: 14.91 ± 1.55											
Lv, 2024^b^	70	I: 16.00 ± 2.00	22/48	Right DLPFC	10 Hz	15	120	45,000	3000	2	+ + ? + +	Treated	II
		C: 15.00 ± 2.00											
Croarkin et al., 2021^a^	103	I: 17.60 ± 2.28	36/67	Right DLPFC	10 Hz	30	120	90,000	3000	6	+ + + + +	Treated	IV
		C: 17.10 ± 2.22											

ROB domains: + low risk of bias; ? some concerns; − high risk of bias. Domains: D1 randomization process, D2 deviations from interventions, D3 missing outcome data, D4 measurement of the outcome, D5 selection of reported result. I: medication type and dosage remain unchanged; II: medication type is fixed but dosage gradually increases; III: real-world study, medication type and dosage change with the condition; IV: no psychotropic medications during the study with the exception of zaleplon, zolpidem, zopiclone, or lorazepam for up to 14 doses. All included studies utilized a parallel-group RCT design. *I* intervention group, *rTMS* combined with antidepressants, *C* control group, sham combined with antidepressant, *TMS* repetitive transcranial magnetic stimulation, *DLPFC* dorsolateral prefrontal cortex, *RCT* randomized controlled trial, *LF* low frequency group, *HF* high frequency group, wk week, *ROB* risk of bias assessed using the Cochrane Risk of Bias tool for randomized trials. ^a^English-language publication; ^b^Chinese-language publication

Participants were recruited exclusively from clinical settings, with ages ranging from 10 to 25; females constituted 60.5% of the sample. Primary outcome data were directly extracted from each study. For the study of Zhao et al., which uniquely designated dual primary outcomes (HAMD-24 and SDS) [30], we prioritized the clinician-rated scale (HAMD-24) over the self-reported measures (SDS) based on minimization of placebo bias in sham-controlled designs. Hence, a total of 14 studies assessed depressive severity using the HAMD. Of these, eight employed the HAMD-24 and six used the HAMD-17. In addition, two studies utilized the BDI-II and SDS, respectively (Table 2). Most patients exhibited mild to moderate depression according to rating scale scores, with only 27% (*n* = 348) of patients classified as severely depressed. Among 16 included studies, seven used low-frequency (1 Hz) stimulation targeting the right DLPFC; six used high-frequency (10 Hz or 15 Hz) targeting the left DLPFC; two employed iTBS targeting the left DLPFC and one study adopted low frequency (LF) and high frequency (HF) intervention in different groups. The mean sample size per study was 81 participants (range: 32–152), with eight trials lasting two weeks, seven studies lasting four weeks and only one study lasting six weeks. With the exception of Croarkin et al. [29] all studies allowed the concurrent use of antidepressant medications. Among these remaining 15 studies, three permitted the use of antidepressant medications with fixed types and dosages. Nine allowed the use of antidepressant medications with fixed types, but the dosage could be gradually increased as needed. The remaining three studies were real-world studies, where patients could follow the medication recommendations of their clinical doctors. All sham groups employed stimulation coils that were visually indistinguishable from those used in the active group, applying the same treatment parameters to stimulate identical areas. To ensure ineffective stimulation, some groups inverted the coils, preventing the stimulation from penetrating the skull and inducing cortical depolarization. Others incorporated a shielding device within the coil to inhibit the delivery of effective magnetic stimulation, thereby achieving the sham stimulation objective. Subjects in the sham groups were able to hear a clear, rhythmic tapping sound, similar to those in the active group. Follow-up data were collected, but were infrequently reported.

**Table 2:** Results of a randomized controlled trial of active stimulation group or sham stimulation group in youth

Studies	Outcome measures	Active stimulation group							Sham stimulation group						
		Baseline, mean ± SD	W1, mea*n* ± SD	W2, mean ± SD	W4/W6, mean ± SD	Response rate, *n* (%)	Remission rate, *n* (%)	Adverse event, *n*	Baseline, mean ± SD	W1, mean ± SD	W2, mean ± SD	W4/W6, mean ± SD	Response rate, %	Remission rate, %	Adverse event, *n*
Zhang, et al., 2024	HAMD-17	22.28 ± 6.30	16.75 ± 6.4	13.05 ± 6.11	NA	9 (31.0)	2 (6.9)	NA	24.17 ± 6.51	17.00 ± 7.66	13.57 ± 8.1	NA	13 (43.3)	3 (10.0)	NA
Zhao, et al., 2023	HAMD-24	28.91 ± 6.70	NA	9.43 ± 4.59	NA	NA	NA	2	25.36 ± 5.43	NA	19.18 ± 5.43	NA	NA	NA	1
Zou, 2023	SDS	57.30 ± 5.96	NA	49.50 ± 10.81	NA	NA	NA	NA	56.93 ± 9.38	NA	54.47 ± 7.93	NA	NA	NA	NA
Wang et al., 2023	HAMD-24	34. 28 ± 5. 60	NA	NA	13.92 ± 4.15	NA	NA	NA	33.63 ± 5.12	NA	NA	16.51 ± 5.34	NA	NA	NA
Lu and Gao, 2020	HAMD-24	27.12 ± 3.24	NA	NA	13.02 ± 3.63	NA	NA	NA	26.67 ± 3.45	NA	NA	19.47 ± 3.96	NA	NA	NA
Qu et al., 2023	HAMD-24	29.16 ± 0.89	27.12 ± 0.88	24.04 ± 1.27	10.44 ± 4.58	15 (50.0)	8 (26.7)	5	29.08 ± 1.03	27.60 ± 0.81	24.96 ± 1.06	17.92 ± 4.97	13 (43.3)	3(10.0)	3
Gu, 2025	HAMD-17	23.65 ± 5.50	12.10 ± 5.56	8.60 ± 4.79	NA	14 (70.0)	11 (55.0)	NA	25.20 ± 7.25	15.70 ± 8.28	12.15 ± 9.26	NA	12 (60.0)	7(35.0)	NA
Chen, et al., 2023	HAMD-17	29.06 ± 5.11	NA	NA	9.10 ± 2.33	24 (47.9)	14 (29.2)	6	27.92 ± 4.71	NA	NA	10.32 ± 2.00	22 (42.9)	7(14.3)	4
Ran et al., 2022	HAMD-17	22.79 ± 3.67	19.78 ± 3.09	15.76 ± 2.95	9.22 ± 2.11	17 (42.5)	14 (35.0)	5	23.16 ± 4.25	21.56 ± 2.86	17.86 ± 3.17	14.63 ± 2.15	15 (37.5)	10(25.0)	9
Liu, 2022	HAMD-24	29.00 ± 5.81	NA	13.63 ± 3.46	NA	NA	NA	NA	31.00 ± 5.90	NA	18.13 ± 3.00	NA	NA	NA	NA
Li, 2022	BDI-II	46.28 ± 12.96	NA	33.72 ± 10.31	27.09 ± 13.53	NA	NA	NA	43.54 ± 15.52	NA	32.62 ± 10.52	29.52 ± 11.56	NA	NA	NA
Fu et al., 2022	HAMD-24	30.47 ± 5.62	NA	NA	14.27 ± 3.14	22 (42.3)	9 (17.3)	3	30.98 ± 5.70	NA	NA	18.05 ± 4.20	18 (34.6)	4(7.7)	0
Gao et al., 2021	HAMD-24	28.69 ± 6.36	NA	13.81 ± 4.46	NA	NA	NA	NA	31.38 ± 9.78	NA	20.56 ± 7.01	NA	NA	NA	NA
Jiao et al., 2024	HAMD-17	LF: 37.27 ± 7.1	NA	LF: 28.09 ± 6.87	LF: 16.22 ± 3.77	LF: 30 (66.7)	NA	LF: 11, HF: 7	36.76 ± 7.79	NA	31.38 ± 7.51	21.31 ± 3.27	23 (51.1)	NA	6
		HF: 36.80 ± 5.51		HF: 25.84 ± 5.38	HF: 15.35 ± 6.23	HF: 32 (71.1)									
Lv, 2024	HAMD-17	22.00 ± 1.02	16.67 ± 1.24	14.67 ± 1.37	NA	NA	NA	NA	21.34 ± 0.82	18.03 ± 0.89	14.80 ± 1.15	NA	NA	NA	NA
Croarkin et al., 2021	HAMD-24	28.80 ± 5.75	NA	NA	18.1 ± 10.91	NA	NA	NA	29.50 ± 6.69	NA	NA	19.20 ± 11.03	NA	NA	NA

*HAMD* Hamilton depression Scale, *SDS* Self-rating Depression Scale, *BDI-II* Beck Depression Inventory-II, *W1* one week, *W2* two weeks, *W4* four weeks, *LF* low frequency group, *HF* high frequency group, *NA* not available

Dropout rates were analyzed across 16 studies. In 12 studies (75%) there were no dropouts in either group. Meta-analysis showed that there was no significant difference in dropout rates between the active and sham groups (RD = 0.00, 95% CI =  – 0.01 to 0.01). Heterogeneity was negligible (*I*^*2*^ = 0%, *P* = 0.98).

**Fig. 2:**
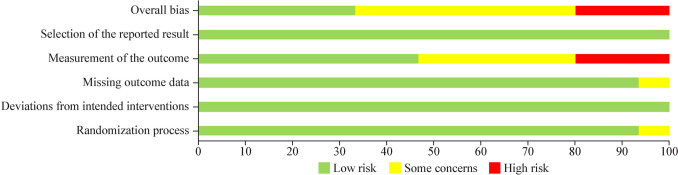
Risk of bias assessment using the Cochrane Risk of Bias tool for randomized trials tools

Risk of study bias is presented in Fig. 2. Overall, the quality of randomized controlled trials (RCTs) in this study was deemed high, with the primary concern being measurement bias due to study participants being aware of the intervention received.

### Intervention efficacy

**Fig. 3:**
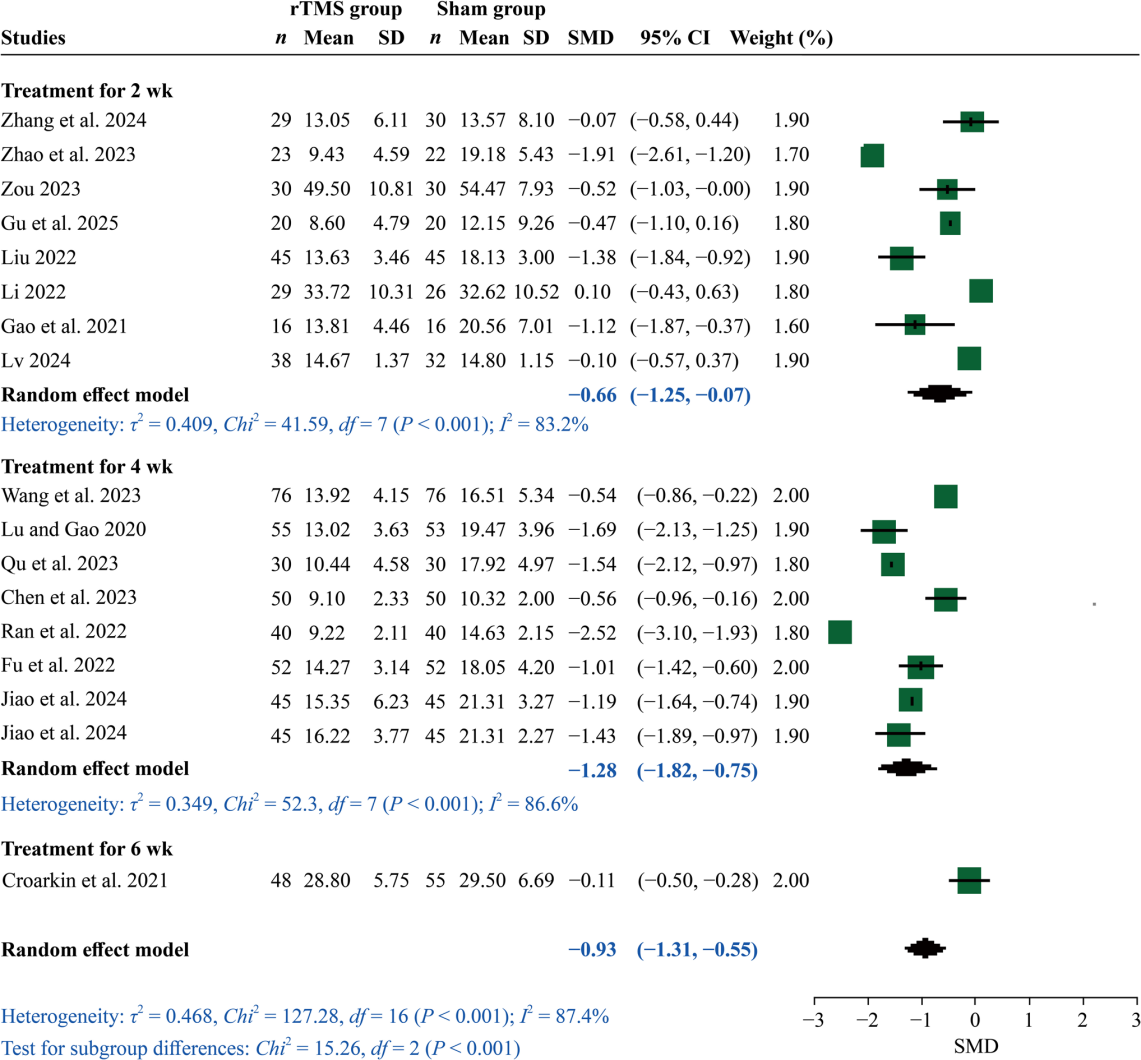
Forest plot of active repetitive transcranial magnetic stimulation intervention versus sham stimulation for the primary outcome of overall symptom severity. *rTMS* repetitive transcranial magnetic stimulation, *SMD* standard mean deviation, *SD* standard deviation, *CI* confidence interval

There were differences in studies reporting depression assessment scores over varying treatment durations. In addition, four studies evaluated treatment efficacy at multiple time points. Therefore we only extracted data from the final time point in each study for use in statistical analysis. Meta-analysis revealed substantial treatment efficacy of active rTMS interventions in improving depressive symptoms in youth (SMD = – 0.93, 95% CI = – 1.31 to – 0.55). Further subgroup analysis showed significant reductions in depression scores at the end of the second week (SMD =  – 0.66, 95% CI = – 1.25 to – 0.07) and persisting to the fourth week (SMD = – 1.28, 95% CI = – 1.82 to – 0.75). This was no longer observed by the end of week six when compared to sham stimulation (Fig. 3). High degree of heterogeneity was observed among the studies included (*I*^2^ = 87.4%), indicating substantial variation in effect sizes. Moreover, sensitivity analysis confirmed the robustness of these findings (Supplementary Fig. 1a). Publication bias was evaluated using funnel plots and Egger’s test; there was no significant bias (*Z* =   – 1.35, *P* = 0.18) (Supplementary Fig. 2a).

Subgroup analyses were generally consistent with the main analysis, demonstrating the efficacy of rTMS across most subgroups compared to sham stimulation. The exception was the real-world study group and “no psychotropic medications” group (Table 3). Further meta-regression analyses identified no significant associations between study characteristics (gender ratio, stimulation frequency, treatment duration, depression severity and medication during intervention) and rTMS efficacy (Table 4). Subgroup analysis by episode chronicity was not feasible due to incomplete reporting; four studies exclusively enrolled first-episode patients and one included mixed cases; 11 failed to specify episode status and no trials focussed solely on recurrent depression.

**Table 3:** Results of subgroup meta-analyses

Subgroups	Number of studies	SMD (95% CI)	Heterogeneity (intra-group)	Heterogeneity (between-groups)
			*I*^*2*^	*P*
Frequency				0.79
TBS	2	– 0.97 (– 2.77, 0.83)	94.0%	
High (10 Hz/15 Hz)	7	– 1.06 (– 1.65, – 0.46)	92.4%	
Low (1 Hz)	8	– 0.81 (– 1.19, – 0.44)	76.6%	
Depression severity				0.13
Mild-moderate	11	– 1.19 (– 1.55, – 0.67)	88.1%	
Severe	6	– 0.62 (– 1.08, – 0.15)	84.4%	
Sham stimulation				
Coil flipping	10	– 0.83 (– 1.29, – 0.36)	89.6%	0.47
Coil shielding	7	– 1.07 (– 1.54, – 0.60)	8.7%	
Pharmacological treatment before rTMS				0.20
Treated	11	– 0.75 (– 1.10, – 0.41)	82.3%	
Untreated	6	– 1.23 (– 1.88, – 0.58)	90.1%	
Medication during intervention				0.01
I	3	– 1.30 (– 2.14, – 0.46)	83.1%	
II	10	– 1.03 (– 1.48, – 0.58)	89.3%	
III	3	– 0.54 (– 1.13, 0.06)	75.5%	
IV	1	– 0.10 (– 0.49, 0.29)	–	

*SMD* standard mean deviation, *I* Medication type and dosage remain unchanged, *II* Medication type is fixed but dosage gradually increases, *III* Real-world study, medication type and dosage change with the condition, *IV* no psychotropic medications during the study with the exception of zaleplon, zolpidem, zopiclone, or lorazepam for up to 14 doses

**Table 4:** Meta-regression results

Variables	Coefficient		SE		95% CI		*P*
Gender ratio	– 0.24		0.45		– 0.64, 1.12		0.59
Frequency	0.38		0.73		– 1.25, 2.13		0.58
Sessions	0.19		0.25		– 0.31, 0.68		0.46
%RMT	0.01		0.02		– 0.02, 0.04		0.49
Treatment duration (wk)	– 0.98		1.16		– 3.25, 1.29		0.40
Depression severity	– 0.76		0.55		– 1.83, 0.31		0.82
Medication during intervention	– 0.09		0.39		– 0.86, 0.67		0.16

*RMT* resting motor threshold, *SE* standard error, *CI* confident interval

**Fig. 4:**
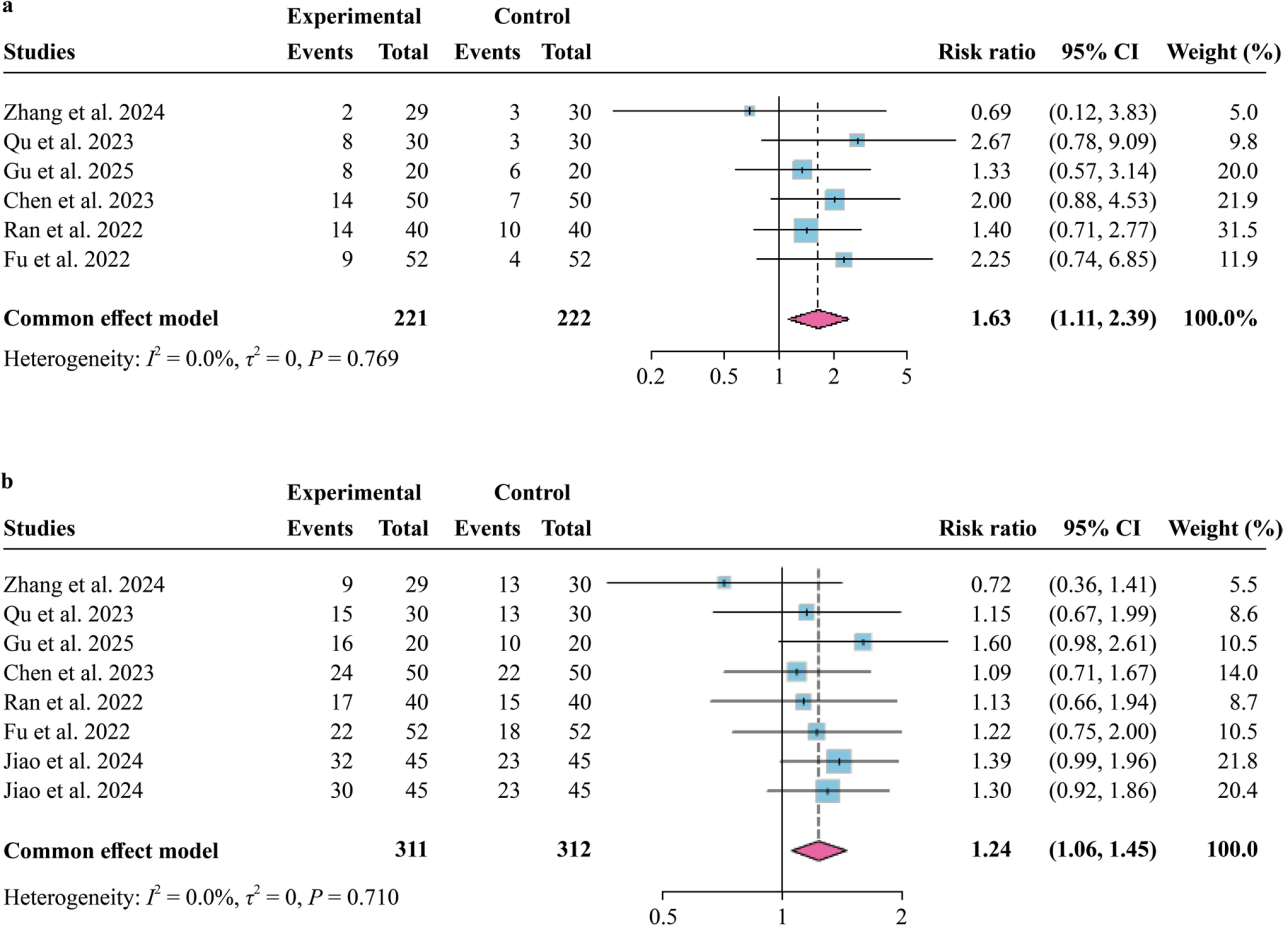
Forest plot of active rTMS intervention versus sham stimulation for the secondary outcome. **a** Remission rate; **b** response rate. *CI* confidence interval

Regarding secondary measures of efficacy, six of 16 studies simultaneously reported response and remission rates; one study only reported response rate [34]. All studies employed the HAMD scale, applying uniform criteria for defining remission and response rates (a reduction of ≥ 75% based on rating scales or a severity level lower than mild was defined as remission and a reduction of ≥ 50% but < 75% was defined as a response). Overall, remission rates were higher in the active rTMS group [risk ratio (RR) = 1.63, 95% CI = 1.11–2.39, *I*^*2*^ = 0%] with an NNT of 10 (95% CI = 4.7‒47.9). Response rates were also significantly different (RR = 1.24, 95% CI = 1.06–1.45, *I*^*2*^ = 0%) when compared to sham stimulation (Fig. 4). Sensitivity and publication bias analyses are presented in Supplementary Fig. 1b and Supplementary Fig. 2b.

### Safety

**Fig. 5:**
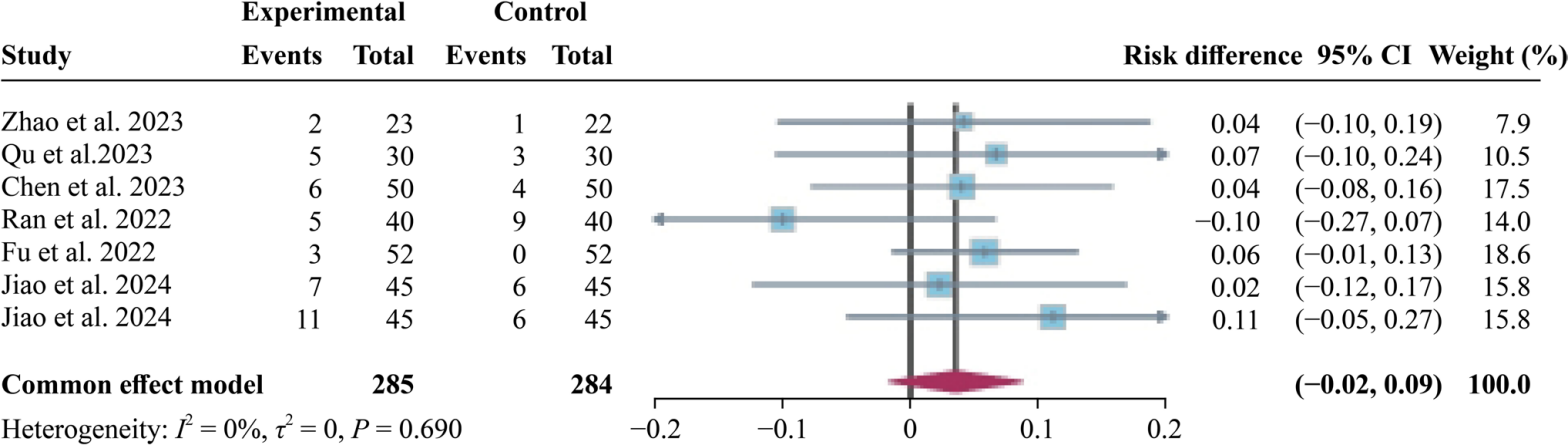
Meta-analysis of adverse events. *CI* confidence interval

Regarding safety, there was no clear difference in adverse events (AEs) for active treatment compared to the sham stimulation group (Fig. 5). No significant heterogeneity was observed across trials (*I*^*2*^ = 0%). Further subgroup analysis by different sham type indicated no significant difference in the incidence of adverse events between the active stimulation and sham stimulation groups (Supplementary Fig. 3). With regard to identification of AEs, the included studies recorded spontaneously reported adverse events and inquired about predetermined symptoms during treatment sessions. Most of the original studies only provided the total number of AE occurrences, with few providing the frequency of specific adverse events. Therefore, we could only count the frequency of occurrence without assigning different weights to different AEs. Common side effects included headache, dizziness, nausea, insomnia and eye pain, with one study reporting suicidal ideation in the active rTMS group. Sensitivity and publication bias analyses are detailed in Supplementary Fig. 1c and Supplementary Fig. 2c.

## Discussion

Our meta-analysis provides the most comprehensive and up-to-date synthesis of evidence incorporating a systematic literature search of all available randomized sham-controlled trials to evaluate the therapeutic efficacy and safety of rTMS in youth depression. This updated iteration of previous meta-analyses [27, 28] incorporates the largest number of studies to date and is enriched with the latest randomized sham-controlled trials from the past three years. We provide robust evidence supporting therapeutic efficacy of rTMS on youth depression, which can be observed as early as in the second week of treatment.

We have identified consistent and statistically significant positive effects of rTMS compared to sham control across the primary outcome and nearly all subgroups, with effect sizes ranging from moderate to large. These findings align with evidence from randomized controlled trials (RCTs) in adults. A meta-analysis by Kedzior et al. [58] reported a pooled effect size of Cohen’s *d* = – 0.55 (95% CI = – 0.75 to – 0.35) for high-frequency rTMS, while Mutz et al. [59] found an odds ratio of 3.75 (95% CI = 2.44–5.75) for response rates. Together, these data indicate that the efficacy of rTMS in alleviating depressive symptoms is comparable between youth and adult populations, regardless of stimulation frequency or concomitant medication. Treatment of youth depression has always been a complex and challenging field. This age group is in a critical period of physical and mental development and drug treatment may bring many side effects, while the efficacy of psychotherapy is difficult to guarantee due to individual patient differences [8]. The results of this study further confirmed the efficacy of rTMS in the treatment of youth with depression, providing a new option for clinical treatment. Notably, the therapeutic trajectory observed in our analysis indicates a rapid onset of benefit; significant symptom reduction emerged by week two and persisted to week four. This pattern mirrors the dose–response relationship described in adult studies, where maximal efficacy typically peaks between weeks 3–4 and is sustained for ≥ 16 weeks [60]. This dose–response relationship can be explained by a previous study that demonstrated long-term rTMS may have lasting efficacy on neural excitability, leading to changes in brain structure and function that promote recovery from depression [61]. Moreover, extended therapeutic courses of rTMS have been shown to prevent relapse in adults with TRD [62, 63]. However, the six-week trial by Croarkin et al., which exclusively targeted treatment-resistant depression (TRD) in youth, revealed a critical divergence as it found no significant separation from sham stimulation at the six-week endpoint (SMD =  – 0.11, 95% CI = – 0.50 to 0.28) [29]. We interpret this null finding through two complementary, non-mutually exclusive hypotheses. First, the study cohort comprised rigorously defined TRD patients [43.7% with an antidepressant treatment record (ATR) of level ≥ 2], a population with distinct neurophysiological alterations that may require more intensive or prolonged rTMS protocols than the 6-week course applied. Second, despite adult data suggesting sustained gains beyond four weeks [64, 65], youth-specific factors—such as dynamic neurodevelopment and a potentially different trajectory of placebo response decay [66]—might shift the optimal therapeutic window earlier, meaning the benefits plateau or decline after 4 weeks in this population. The protocol in Croarkin et al. may thus have been either insufficient for the severe TRD cohort or misaligned with the neurodevelopmental timing of peak response in youth.

A conventional rTMS treatment course is recommended to last for 4‒6 weeks [67], however, the evidence of TMS in youth is limited. The substantial statistical heterogeneity observed across studies (*I*^*2*^ = 87.4%) primarily stems from three key methodological sources. First, variability in stimulation parameters, including fundamental differences in frequency (e.g., excitatory 10 Hz vs. inhibitory 1 Hz protocols) and modality (e.g., TBS, which induces more pronounced motor cortex excitability changes compared to conventional LF or HF rTMS protocols [68, 69]), directly influences neurophysiological outcomes. Second, significant variation in treatment dose was evident, with total pulses per session ranging widely from 600 to 3000 (representing a five-fold difference). Of note, meta-regression analysis revealed no linear correlation between pulse count and therapeutic efficacy, suggesting potential youth-specific dose–response thresholds or nonlinear neuroadaptation. Finally, considerable clinical heterogeneity existed across study populations regarding disease characteristics (e.g., TRD vs. first-episode patients, presence or absence of psychotic features) and concomitant pharmacotherapy (e.g., rTMS monotherapy vs. combinations with antidepressants or antipsychotics). We did not find a significant correlation between treatment dose-related parameters (such as the number of pulses, duration and number of sessions) and therapeutic efficacy, making it difficult to recommend optimal treatment duration for adolescents and young people. Currently, there is no unified standard for treatment parameters and duration of TMS future RCTs are needed to explore the treatment parameters, further optimize TMS parameters and explore the optimal treatment duration to improve both therapeutic efficacy and safety.

In addition, the rate of remission, a commonly used indicator in clinical settings to evaluate the effectiveness, provides additional support for the efficacy of rTMS. Although there is a lack of data on remission or response rates at the end of two weeks of treatment, we did find that the active rTMS was associated with significantly higher remission rates than sham stimulation at the end of four weeks of treatment; there was a clinically relevant NNT of 10. This effect is comparable to the meta-analysis with high-frequency rTMS in adults with depression, revealing an NNT of 8 for the remission outcome [70]. Although an NNT of less than or equal to 10 is generally considered to be clinically significant [43], the wide confidence interval obtained in this study suggests caution in interpreting this result. More primary studies are needed to explore this issue further and to better inform clinical practice. The response rate remained significantly higher in the TMS group compared to sham stimulation, consistent with prior evidence. Prior meta-analyses in both adults and adolescents have reported higher response rates with active rTMS compared to sham stimulation [28, 70]. However, limited sample sizes and heterogeneous treatment protocols precluded delineation of potentially heightened placebo effects in youth populations [66] and meaningful subgroup analyses to establish optimal therapeutic regimens for this demographic. In summary, the results of remission and response rates of rTMS for depression in youth require further investigation.

Consistent with previous studies, our analysis shows that rTMS has a similar tolerability profile and transient side effects to sham stimulation [27, 28, 71], including headaches, dizziness and nausea, thereby reinforcing safety for clinical application. The observed near-zero dropout rates (75% studies with no attrition) and nonsignificant RD = 0.00 also suggest excellent tolerability of rTMS protocols in youth populations. Nonetheless, it is important to recognize the potential for serious adverse reactions, such as seizures and suicidal ideation. Rossi et al. noted that rTMS can trigger paroxysmal hypersynchronous neural discharges, which may lead to seizures, particularly in youth with heightened cortical excitability [72]. Although the incidence of TMS-related seizures in children and adolescents is low at 0.62% [48, 71], there is still a need for further research specifically focusing on this problem. Seizures can cause significant psychological and physical harm and this can affect treatment adherence. Thorough pre-treatment screening for seizure risk factors, obtaining informed consent and ensuring professional supervision during treatment are crucial. Another severe side effect is suicidal ideation. In this review, none of the studies reported adverse effects related to suicidal ideation. However, a previous large-scale adolescent study documented such occurrences [29], which might be attributed to the inclusion of a sample with treatment-resistant depression and the absence of combined pharmacotherapy during treatment. This suggests that the occurrence of suicidal ideation may be related to the natural progression of the disease rather than rTMS treatment. Huang et al. previously reported that neuronavigation-guided high-frequency rTMS can improve suicidal ideation and depressive symptoms in depressed patients (13‒45 years old) within one week [48]. However, a recent meta-analysis suggested that in adolescents and/or adults aged 16 years or older diagnosed with unipolar or bipolar depression, the efficacy of rTMS for improving suicidal ideation in patients with TRD is inconclusive [73]. Therefore, the relationship between rTMS treatment and the presence of suicidal ideation remains unclear and requires further study.

There are several limitations to this study. First, our analysis was restricted to the immediate post-treatment efficacy of TMS, as few studies reported long-term follow-up data. This limits the ability to assess the durability of long-term antidepressant efficacy over extended periods and potential long-term tolerability or adverse effects associated with long-term use [74]. However, a pioneering study from Zhang et al. found that the efficacy of rTMS in adolescents remained significant until week four follow-up in real world study [22]. Moreover, previous small-sample self-control studies suggested continuous improvement of depression score over a longer period without significant side effects [17, 23]. Hence, we emphasize the need for future, large-scale sham-controlled studies with long-term follow-up in youth with depression to comprehensively examine efficacy and adverse events. Secondly, our analysis did not discriminate rTMS as a monotherapy or an augmentation strategy. This is partly due to rTMS not being recommended as a first-line treatment for adolescent depression, leading to most studies involving rTMS combined with antidepressant medication. However, in adult studies, there are reports showing that rTMS is equally effective whether used as an enhancement strategy or as a monotherapy for MDD [70, 75]. Thirdly, our meta-analysis included studies evaluating the efficacy of the two most popular rTMS treatment protocols on depression; conventional rTMS (low-frequency and high-frequency) and iTBS, leaving the efficacy of other TMS modalities in youth unexplored. Fourth, due to limited data, we could not assess the efficacy of rTMS in youth with TRD. Preliminary studies investigating the effectiveness of rTMS in TRD have yielded conflicting results. For instance, Croarkin et al. reported no significant differences in symptom severity between the rTMS and sham groups after a six-week intervention [29]. However, two small-sample open trials demonstrated significant reductions in depressive symptom severity following rTMS treatment [76, 77]. The efficacy of rTMS in youth with TRD remains inconclusive, necessitating further investigation through additional randomized controlled trials with sham controls. Moreover, subgroup analysis of severe depression yielded negative outcomes, an observation based on only two trials, necessitating cautious interpretation. In addition, the predominance of unclassified episode status limits chronicity-specific inferences. Fifth, our study included subjects spanning childhood to early adulthood, which results in significant physiological and psychological differences among individuals, such as brain development, cognitive function and emotional regulation ability. Studies indicate that neuropsychological development may contribute to the inconsistency in the results of rTMS treatment for adolescent depression. Major changes in brain structure and function can lead to an imbalance between excitation and inhibition, alter cortical plasticity and connectivity and affect the efficiency of information exchange between key brain areas involved in emotion processing [38]. However, since no original studies compared efficacy across different age groups and age ranges in the included studies were similar (close average ages of subjects in each study), we were unable to stratify by age, especially in 10–14 age groups to investigate differences in the efficacy of transcranial magnetic stimulation across ages. We encourage future large-scale randomized controlled studies in adolescent populations to further explore efficacy by stratifying into different developmental stages. Finally, it is noteworthy that all the studies we included were conducted in China; this might be influenced by the affordability of Chinese health insurance policies. While the efficacy and safety of rTMS have been confirmed in Australia [21], the United States [78] and Canada [76], generalizing the findings of this study to other ethnic groups should be done cautiously.

In conclusion, this systematic review and meta-analysis of randomized sham-controlled trials underscores the potential of rTMS as a viable and efficacious treatment for 10‒25-year-olds (youth) suffering from depression. Overall, this meta-analysis provides high-certainty evidence to support consideration of rTMS as a safe and effective treatment for youths with depression. Future research should focus on long-term outcomes, the role of rTMS in TRD, more precise and tailored approach to treatment and an exploration of its use across diverse populations to further validate its therapeutic potential and safety profile.

## Supplementary Information

Below is the link to the electronic supplementary material.

Supplementary file 1 (PDF 1322 KB)

## Data Availability

All data generated or analyzed during this study are included in this published article.
