# Supplementary material for: Efficacy and safety of repetitive transcranial magnetic stimulation in youth with depression: a systematic review and meta-analysis of randomized sham-controlled trials
Source: World J Pediatr. 2025 Oct 25;21(12):1258–74. doi: 10.1007/s12519-025-00983-7 (PMC12678628; doi:10.1007/s12519-025-00983-7)
Supplement: Supplementary file 1 — (PDF 1322 KB) [file 12519_2025_983_MOESM1_ESM.pdf]

Supplementary Material

1 Supplementary search strategy

Literature search was no language limitation

- Pubmed n=150

"depression"[Title/Abstract] OR "depress*"[Title/Abstract] OR "depressive disorder"[Title/Abstract] OR "depression"[MeSH Terms]

AND

"adolescent"[MeSH Terms] OR "adolescen*"[Title/Abstract] OR "Teens"[Title/Abstract] OR "Teen"[Title/Abstract] OR "Teenagers"[Title/Abstract] OR "Teenager"[Title/Abstract] OR "Youth"[Title/Abstract] OR "Youths"[Title/Abstract]

AND

"transcranial magnetic stimulation"[MeSH Terms] OR "transcranial magnetic stimulation"[Title/Abstract] OR "TMS"[Title/Abstract] OR "iTBS"[Title/Abstract] OR " theta burst stimulation"[Title/Abstract] OR "repetitive transcranial magnetic stimulation"[Title/Abstract] OR "rTMS"[Title/Abstract]

AND

"randomized controlled trials"[MeSH Terms] OR "controlled clinical trial"[Title/Abstract] OR "random allocation"[Title/Abstract] OR "double-blind"[Title/Abstract] OR "placebo"[Title/Abstract] OR "randomly"[Title/Abstract] OR "randomized"[Title/Abstract] OR "clinical trial"[Title/Abstract] OR "trial"[Title/Abstract] OR "RCT"[Title/Abstract]

- Web of science n=554

TS=depression OR depress* OR depressive disorder

AND

TS=adolescent OR adolescen* OR Teens OR Teen OR Teenagers OR Teenager OR Youth OR Youths

AND

TS=transcranial magnetic stimulation OR TMS OR iTBS OR theta burst stimulation OR repetitive transcranial magnetic stimulation OR rTMS OR magnetic stimulation

AND

TS=randomized controlled trials OR controlled clinical trial OR random allocation OR double-blind OR placebo OR randomly OR randomized OR clinical trial OR trial OR RCT OR randomly

- Embasen n=146

#13. #3 AND #6 AND #9 AND #12

#12. #10 OR #11

#11. 'randomized controlled trial':ab,ti OR 'controlled clinical trial':ab,ti OR 'random allocation':ab,ti OR 'double blind':ab,ti OR placebo:ab,ti OR randomly:ab,ti OR randomized:ab,ti OR 'clinical trial':ab,ti OR trial:ab,ti OR rct:ab,ti

#10. 'randomized controlled trial'/exp

#9. #7 OR #8

#8. 'transcranial magnetic stimulation':ab,ti OR tms:ab,ti OR itbs:ab,ti OR 'repetitive transcranial magnetic stimulation':ab,ti OR 'theta burst stimulation':ab,ti AND rtms:ab,ti

#7. 'transcranial magnetic stimulation'/exp

#6. #4 OR #5

#5. depression:ab,ti OR 'depressive disorder':ab,ti OR depress*:ab,ti

#4. 'depression'/exp

#3. #1 OR #2

#2. 'adolescent'/exp

#1. teenager:ab,ti OR adolescent:ab,ti

- Cochrane library n=209

#1 adolescent :ab,ti,kw OR Adolescents :ab,ti,kw OR Teens :ab,ti,kw OR Teen :ab,ti,kw OR Teenagers :ab,ti,kw OR Teenager :ab,ti,kw OR Youth :ab,ti,kw OR Youths :ab,ti,kw

#2 Transcranial Magnetic Stimulation :ab,ti,kw OR iTBS :ab,ti,kw OR rTMS :ab,ti,kw OR TMS :ab,ti,kw OR theta burst stimulation :ab,ti,kw

#3 depression :ab,ti,kw OR depress* :ab,ti,kw OR depressive disorder :ab,ti,kw

#4 Randomized Controlled Trial :ab,ti,kw OR controlled clinical trial :ab,ti,kw OR random allocation :ab,ti,kw OR double-blind :ab,ti,kw OR placebo :ab,ti,kw OR randomly :ab,ti,kw OR randomized :ab,ti,kw OR clinical trial :ab,ti,kw OR trial :ab,ti,kw OR RCT :ab,ti,kw

#5 #1 AND #2 AND #3 AND #4

- Scopusn n=263

TITLE-ABS-KEY "randomized controlled trial" OR TITLE-ABS-KEY "controlled clinical trial" OR TITLE-ABS-KEY "random allocation" OR TITLE-ABS-KEY double-blind OR TITLE-ABS-KEY placebo OR TITLE-ABS-KEY randomly OR TITLE-ABS-KEY randomized OR TITLE-ABS-KEY "clinical trial" OR TITLE-ABS-KEY trial OR TITLE-ABS-KEY rct AND TITLE-ABS-KEY depression OR TITLE-ABS-KEY depress* OR TITLE-ABS-KEY "depressive disorder" AND TITLE-ABS-KEY "transcranial magnetic stimulation" OR TITLE-ABS-KEY itbs OR TITLE-ABS-KEY rtms OR TITLE-ABS-KEY tms OR TITLE-ABS-KEY "theta burst stimulation" AND TITLE-ABS-KEY adolescent OR TITLE-ABS-KEY adolescents OR TITLE-ABS-KEY teens OR TITLE-ABS-KEY teen OR TITLE-ABS-KEY teenagers OR TITLE-ABS-KEY teenager OR TITLE-ABS-KEY youth OR TITLE-ABS-KEY youths

- PsycInfon n=65

S1.SU depression OR XB depress OR XB depressive disorder

S2.SU adolescent OR XB Teens OR XB Teen OR XB Teenagers OR XB Teenager OR XB Youth OR XB Youths

S3.SU transcranial magnetic stimulation OR XB Transcranial Magnetic Stimulation OR XB iTBS OR XB rTMS OR XB TMS OR XB theta burst stimulation OR XB repetitive transcranial magnetic stimulation

S4.SU randomized controlled trials OR XB Randomized Controlled Trial OR XB controlled clinical trial OR XB random allocation OR XB double-blind OR XB placebo OR XB randomly OR XB randomized OR XB clinical trial OR XB RCT

S5. [S1] AND [S2] AND [S3] AND [S4]

- ProQuest Dissertations and Theses PQDT n=30

S1.abstract(adolescent) OR abstract(Teen) OR abstract(Teenager) OR abstract(Youth)

S2.abstract(Transcranial Magnetic Stimulation) OR abstract(iTBS) OR abstract(rTMS) OR abstract(TMS) OR abstract(theta burst stimulation)

S3.abstract(depression) OR abstract(depress*) OR abstract(depressive disorder)

S4.abstract(Randomized Controlled Trial) OR abstract(controlled clinical trial) OR abstract(random allocation) OR abstract(double-blind) OR abstract(placebo) OR abstract(randomly) OR abstract(randomized) OR abstract(clinical trial) OR abstract(trial) OR abstract(RCT)

S5.[S1] AND [S2] AND [S3] AND [S4]

- CNKI n=40

（SU = '青少年' OR TKA = '青少年' OR TKA = '儿童'）AND（SU = '抑郁障碍' OR TKA = '抑郁障碍' OR TKA = '抑郁症'）AND（SU = '经颅磁刺激' OR TKA = '重复经颅磁刺激' OR TKA = 'TMS' OR TKA = 'TBS' OR TKA = 'rTMS' OR TKA = '爆发性θ波刺激'）AND（SU = '随机对照试验' OR TKA = '随机对照试验' OR TKA = '随机' OR TKA = '对照试验' OR TKA = '盲法' OR TKA = '随机分配'）

- WANFANG n=8

(主题:(青少年) or 题名或关键词:(儿童) or 题名或关键词:(青少年)) and (主题:(抑郁症) or 题名或关键词:(抑郁障碍) or 题名或关键词:(抑郁症)) and (主题:(经颅磁刺激) or 题名或关键词:(重复经颅磁刺激) or 题名或关键词:(TMS) or 题名或关键词:(TBS) or 题名或关键词:(θ爆发式刺激)) and (主题:(随机对照试验) or 题名或关键词:(随机) or 题名或关键词:(对照试验) or 题名或关键词:(盲法))

2 Supplementary Figures

a.Standard mean deviation of depression score sensitivity analyses results

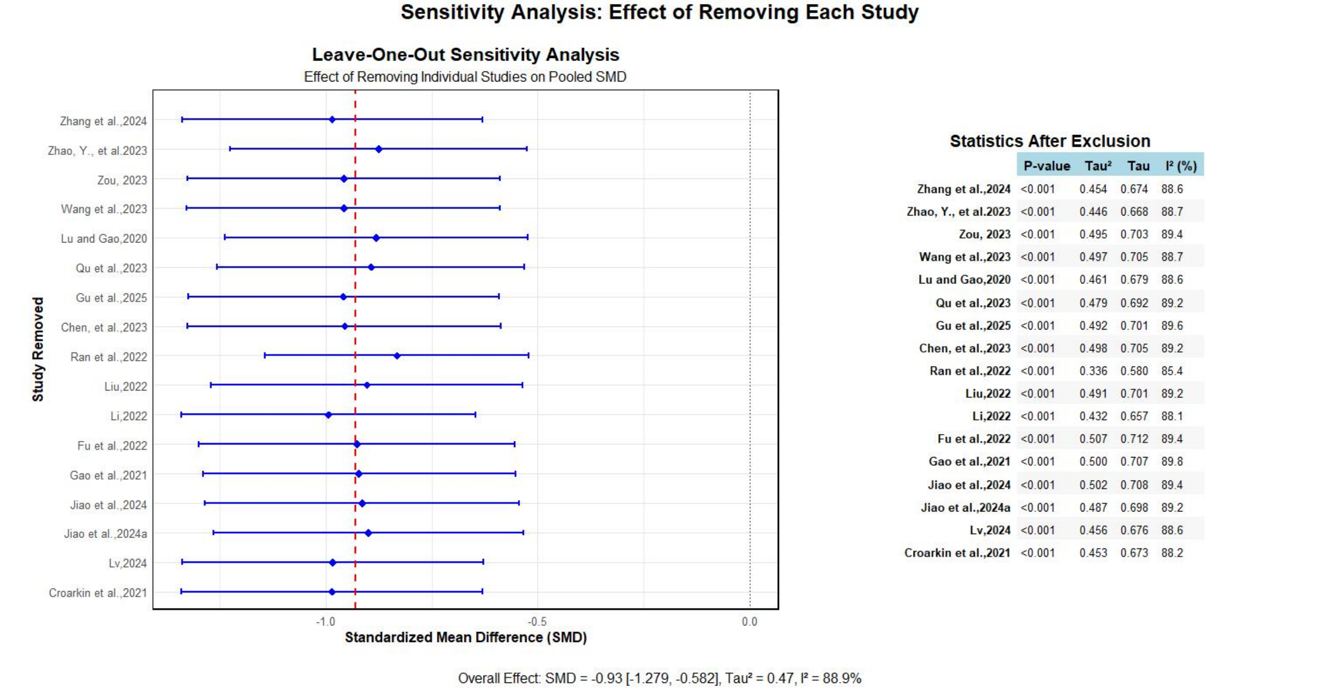

b.Results of sensitivity analyses in remission rate (**i**) and response rate (**ii**)

i

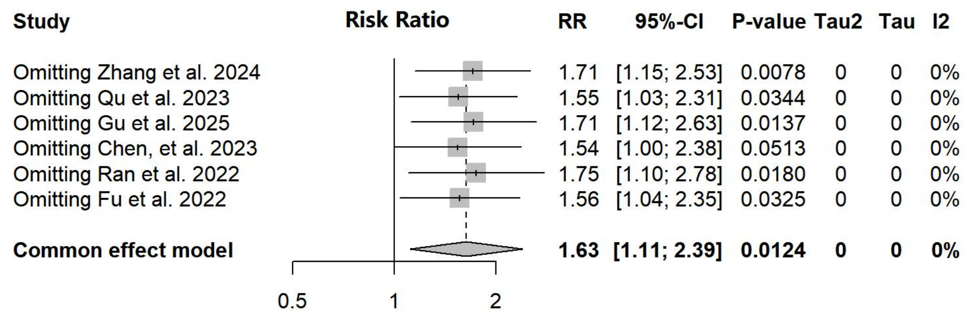

ii

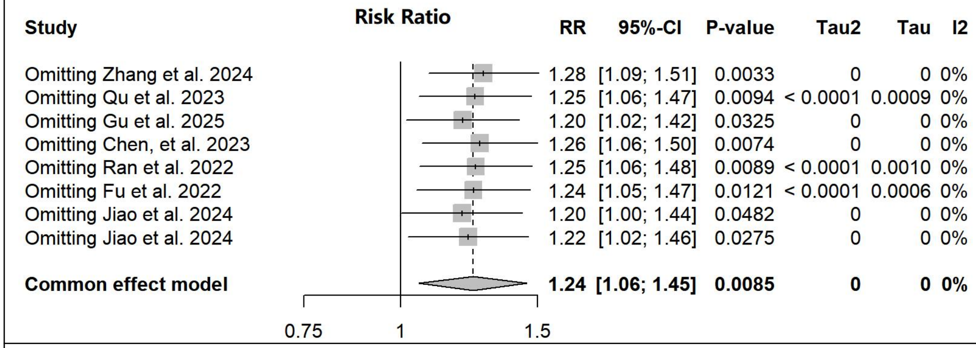

c.Results of sensitivity analyses in adverse events

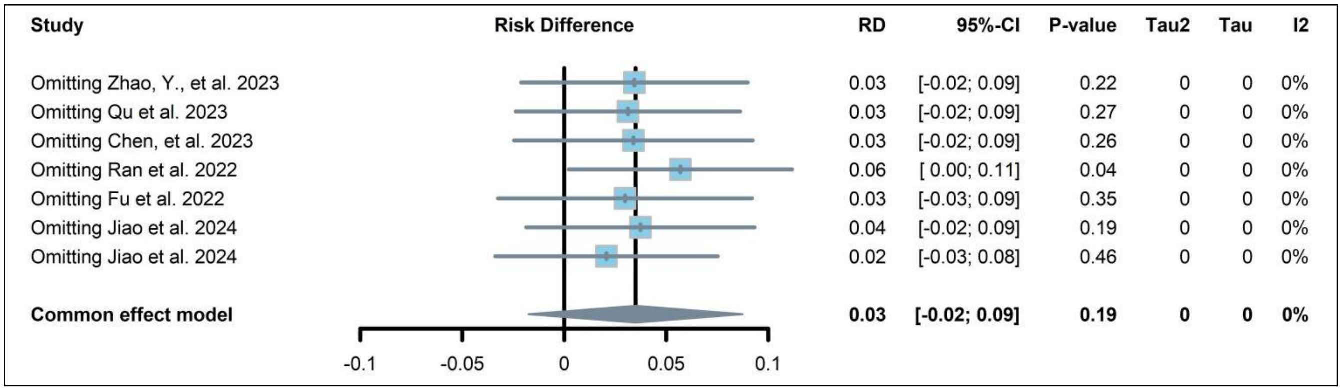

Supplementary Fig. 1 Results of sensitivity analysis by leaving studies one by one

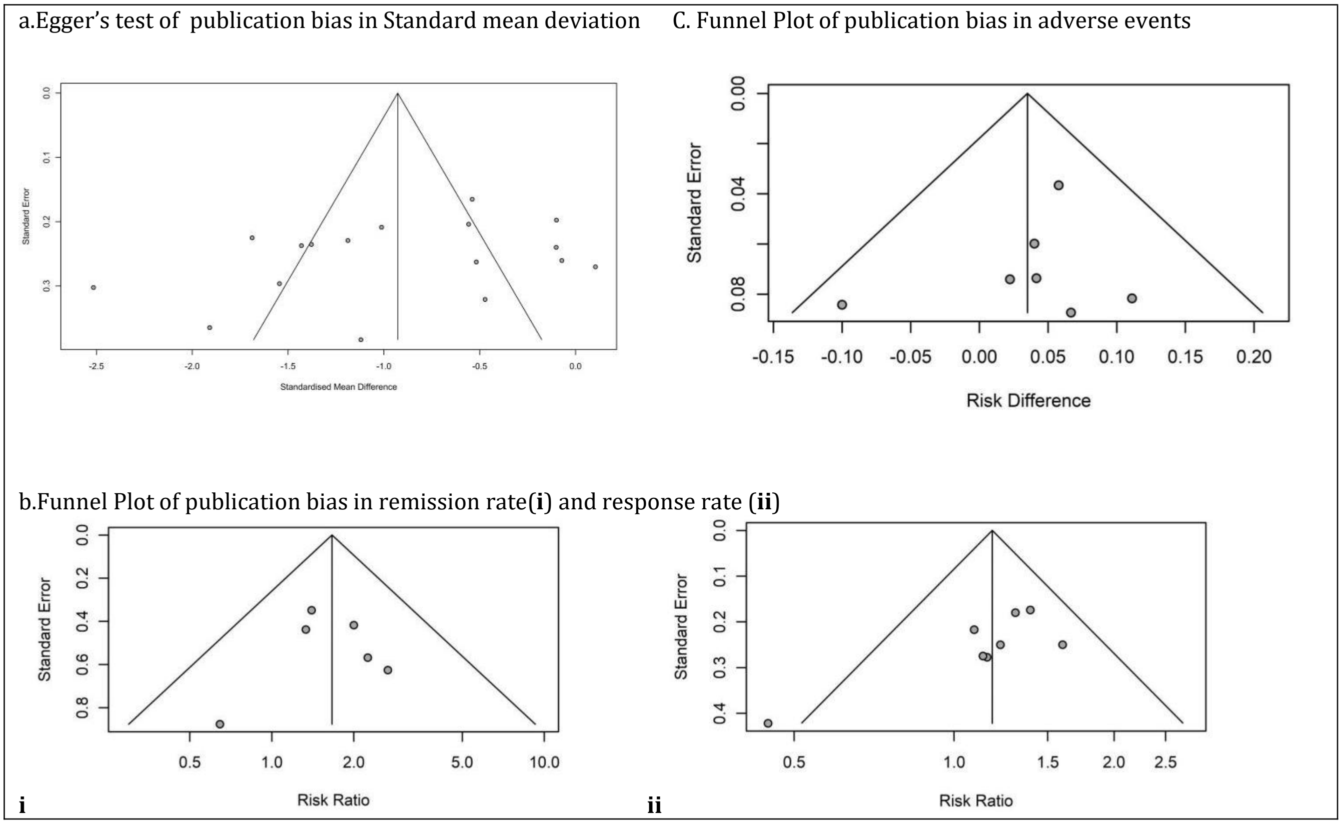

Supplementary Fig 2. Results of publication bisas

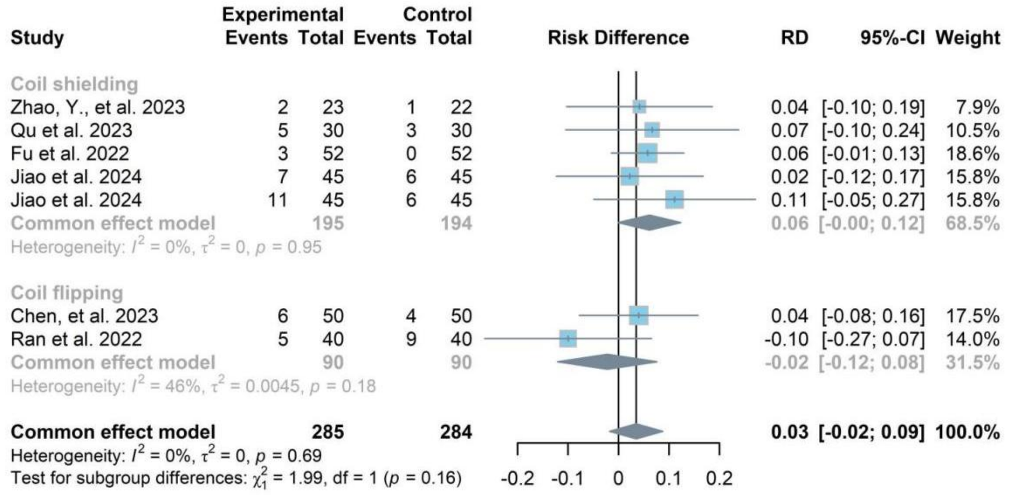

Supplementary Fig. 3 Meta-analysis result of adverse events in different sham type groups

Graphical abstract

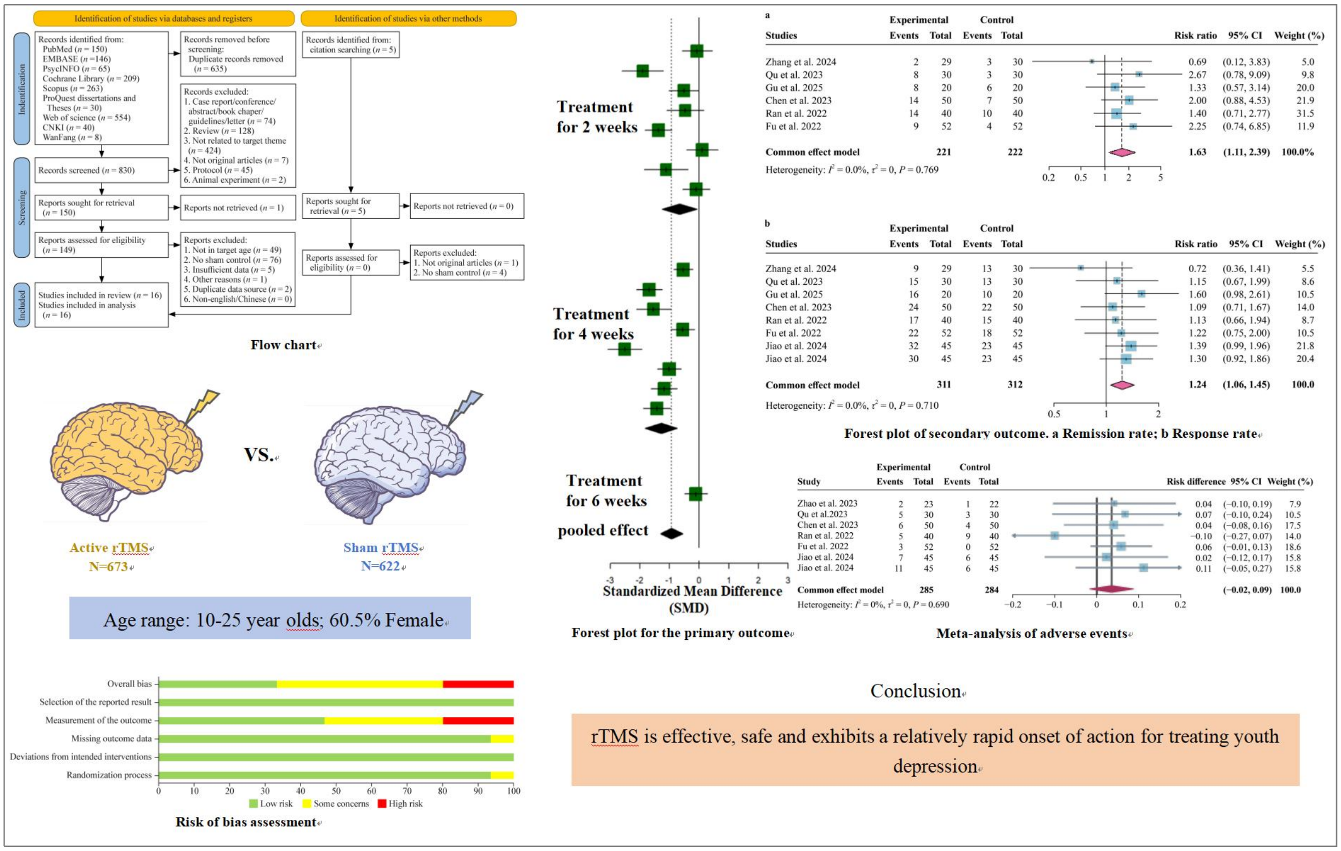

科研诚信承诺书

稿件题目： Efficacy and safety of repetitive transcranial magnetic stimulation in youth with depression: A systematic review and meta-analysis of randomized sham-controlled trials

稿件编号： WJOP-D-25-00784-R1

通讯作者： 黄颐　单位及部门： 四川大学华西医院心理卫生中心

本人在此郑重承诺：

1）本文章涉及的研究不存在科研不端行为。

2）本文章不存在重复发表情况（**包括未在中文期刊发表过**）。

3）本文章不存在一稿多投情况。

4）本文章不存在数据篡改、捏造及图片造假等情况。

5）本人不存在违背中共中央办公厅、国务院办公厅《关于进一步加强科研诚信建设的若干意见》规定和其他科研诚信要求，以及国家、学校及学院等其他相关规定的行为。

通讯作者签名：

（须盖单位科研管理部门公章）

2025　年 8 月 8 日

Section and Topic	Item #	Checklist item	Location where item is reported
**TITLE**			
Title	1	Identify the report as a systematic review.	P1
**ABSTRACT**			
Abstract	2	See the PRISMA 2020 for Abstracts checklist.	P1
**INTRODUCTION**			
Rationale	3	Describe the rationale for the review in the context of existing knowledge.	P3-5
Objectives	4	Provide an explicit statement of the objective(s) or question(s) the review addresses.	P5
**METHODS**			
Eligibility criteria	5	Specify the inclusion and exclusion criteria for the review and how studies were grouped for the syntheses.	P7
Information sources	6	Specify all databases, registers, websites, organisations, reference lists and other sources searched or consulted to identify studies. Specify the date when each source was last searched or consulted.	P6
Search strategy	7	Present the full search strategies for all databases, registers and websites, including any filters and limits used.	P6
Selection process	8	Specify the methods used to decide whether a study met the inclusion criteria of the review, including how many reviewers screened each record and each report retrieved, whether they worked independently, and if applicable, details of automation tools used in the process.	P7-8
Data collection process	9	Specify the methods used to collect data from reports, including how many reviewers collected data from each report, whether they worked independently, any processes for obtaining or confirming data from study investigators, and if applicable, details of automation tools used in the process.	P8
Data items	10a	List and define all outcomes for which data were sought. Specify whether all results that were compatible with each outcome domain in each study were sought (e.g. for all measures, time points, analyses), and if not, the methods used to decide which results to collect.	P8
	10b	List and define all other variables for which data were sought (e.g. participant and intervention characteristics, funding sources). Describe any assumptions made about any missing or unclear information.	P8
Study risk of bias assessment	11	Specify the methods used to assess risk of bias in the included studies, including details of the tool(s) used, how many reviewers assessed each study and whether they worked independently, and if applicable, details of automation tools used in the process.	P8-9
Effect measures	12	Specify for each outcome the effect measure(s) (e.g. risk ratio, mean difference) used in the synthesis or presentation of results.	P8
Synthesis methods	13a	Describe the processes used to decide which studies were eligible for each synthesis (e.g. tabulating the study intervention characteristics and comparing against the planned groups for each synthesis (item #5)).	P9-10
	13b	Describe any methods required to prepare the data for presentation or synthesis, such as handling of missing summary statistics, or data conversions.	NA
	13c	Describe any methods used to tabulate or visually display results of individual studies and syntheses.	P8
	13d	Describe any methods used to synthesize results and provide a rationale for the choice(s). If meta-analysis was performed, describe the model(s), method(s) to identify the presence and extent of statistical heterogeneity, and software package(s) used.	P8
	13e	Describe any methods used to explore possible causes of heterogeneity among study results (e.g. subgroup analysis, meta-regression).	P9
	13f	Describe any sensitivity analyses conducted to assess robustness of the synthesized results.	P9

Section and Topic	Item #	Checklist item	Location where item is reported
Reporting bias assessment	14	Describe any methods used to assess risk of bias due to missing results in a synthesis (arising from reporting biases).	NA
Certainty assessment	15	Describe any methods used to assess certainty (or confidence) in the body of evidence for an outcome.	P9
**RESULTS**			
Study selection	16a	Describe the results of the search and selection process, from the number of records identified in the search to the number of studies included in the review, ideally using a flow diagram.	P10
	16b	Cite studies that might appear to meet the inclusion criteria, but which were excluded, and explain why they were excluded.	NA
Study characteristics	17	Cite each included study and present its characteristics.	P10-12
Risk of bias in studies	18	Present assessments of risk of bias for each included study.	P12
Results of individual studies	19	For all outcomes, present, for each study: (a) summary statistics for each group (where appropriate) and (b) an effect estimate and its precision (e.g. confidence/credible interval), ideally using structured tables or plots.	P12-14
Results of syntheses	20a	For each synthesis, briefly summarise the characteristics and risk of bias among contributing studies.	P12-13
	20b	Present results of all statistical syntheses conducted. If meta-analysis was done, present for each the summary estimate and its precision (e.g. confidence/credible interval) and measures of statistical heterogeneity. If comparing groups, describe the direction of the effect.	P12-13
	20c	Present results of all investigations of possible causes of heterogeneity among study results.	P12-13
	20d	Present results of all sensitivity analyses conducted to assess the robustness of the synthesized results.	P12-13
Reporting biases	21	Present assessments of risk of bias due to missing results (arising from reporting biases) for each synthesis assessed.	NA
Certainty of evidence	22	Present assessments of certainty (or confidence) in the body of evidence for each outcome assessed.	P12-14
**DISCUSSION**			
Discussion	23a	Provide a general interpretation of the results in the context of other evidence.	P15
	23b	Discuss any limitations of the evidence included in the review.	P20
	23c	Discuss any limitations of the review processes used.	NA
	23d	Discuss implications of the results for practice, policy, and future research.	P16-20
**OTHER INFORMATION**			
Registration and protocol	24a	Provide registration information for the review, including register name and registration number, or state that the review was not registered.	P5
	24b	Indicate where the review protocol can be accessed, or state that a protocol was not prepared.	P5
	24c	Describe and explain any amendments to information provided at registration or in the protocol.	NA
Support	25	Describe sources of financial or non-financial support for the review, and the role of the funders or sponsors in the review.	title page
Competing	26	Declare any competing interests of review authors.	title page

Section and Topic	Item #	Checklist item	Location where item is reported
interests			
Availability of data, code and other materials	27	Report which of the following are publicly available and where they can be found: template data collection forms; data extracted from included studies; data used for all analyses; analytic code; any other materials used in the review.	title page

From: Page MJ, McKenzie JE, Bossuyt PM, Boutron I, Hoffmann TC, Mulrow CD, et al. The PRISMA 2020 statement: an updated guideline for reporting systematic reviews. BMJ 2021;372:n71. doi: 10.1136/bmj.n7
